# Idiopathic Hypercalciuria: A Comprehensive Review of Clinical Insights and Management Strategies

**DOI:** 10.7759/cureus.81778

**Published:** 2025-04-05

**Authors:** Dayanidhi Meher, Vishal Agarwal, Sambit Das, Arun Choudhury, Devadarshini Sahoo, Sandeep K Sahu, Binod Prusty, Bijay Das

**Affiliations:** 1 Endocrinology, Diabetes and Metabolism, Kalinga Institute of Medical Sciences, Bhubaneswar, IND

**Keywords:** 24-hour urinary calcium, idiopathic hypercalciuria, nephrocalcinosis (nc), osteoporosis, recurrent kidney stones

## Abstract

Idiopathic hypercalciuria (IH) is a metabolic condition characterized by excessive calcium excretion in urine without identifiable secondary causes, such as hyperparathyroidism or malignancy. It is a significant clinical entity due to its association with kidney stones, nephrocalcinosis, and osteoporosis, leading to reduced quality of life and long-term complications. This comprehensive review discusses the pathophysiology, clinical manifestations, diagnostic strategies, and management approaches for IH. The disorder arises from a multifaceted interplay of renal, intestinal, and skeletal factors. Impaired renal tubular calcium reabsorption, heightened intestinal calcium absorption, and increased bone resorption are key contributors to its pathogenesis. Genetic predispositions, including mutations in calcium-regulating receptors and transporters, further complicate its etiology. Patients often present with kidney stones, bone pain, or reduced bone mineral density, although asymptomatic cases are not uncommon. Diagnosing IH requires a thorough evaluation to exclude secondary causes, with 24-hour urinary calcium excretion serving as a crucial diagnostic marker. Management focuses on mitigating complications and improving quality of life through hydration, dietary modifications, and pharmacological therapy. Thiazide diuretics are the cornerstone of treatment, effectively reducing urinary calcium levels and preventing stone formation. Adjunctive measures include citrate supplementation and lifestyle interventions such as weight management and adequate physical activity. For patients with severe nephrolithiasis or nephrocalcinosis, surgical intervention may be necessary. Despite significant advancements, IH remains a diagnostic and therapeutic challenge due to its diverse clinical presentations and underlying mechanisms. A multidisciplinary approach, incorporating tailored medical and dietary strategies, is essential for optimal management. Future research into its genetic and molecular basis holds promise for developing more targeted interventions and improving patient outcomes. This review aims to provide a practical, up-to-date guide for clinicians managing this complex yet common metabolic disorder.

## Introduction and background

Excessive calcium excretion in the urine without a known underlying cause, such as hyperparathyroidism, cancer, or an excess of vitamin D, is known as idiopathic hypercalciuria (IH). As IH is associated with kidney stones, nephrocalcinosis, and osteoporosis, it is a major clinical challenge for endocrinologists.

A daily calcium excretion of more than 300 mg in males and more than 250 mg in females is referred to as hypercalciuria. Most of the time, there are no known etiological causes of hypercalciuria, and blood calcium levels are normal. The condition is then regarded as idiopathic (primary). Approximately 10% of the general population and 40-50% of patients with calcium-based kidney stones have IH. This entity needs to be differentiated from systemic causes of hypercalciuria (secondary) caused by several diseases, including paraneoplastic syndromes, sarcoidosis, and primary hyperparathyroidism [1].

IH is a condition that affects individuals across all age groups, from children to older adults. While some individuals remain asymptomatic, others experience recurrent kidney stones, nephrocalcinosis, bone pain, or fractures. The exact cause of IH remains elusive, but it is widely accepted that a combination of genetic, dietary, and environmental factors contributes to its development. Family studies suggest a strong hereditary component, with first-degree relatives of affected individuals having a significantly higher risk of developing hypercalciuria than the general population. Moreover, lifestyle and dietary factors, such as high sodium intake and low calcium consumption, can exacerbate urinary calcium loss, further influencing disease progression [2].

The consequences of IH extend beyond kidney stone formation. Chronic hypercalciuria has been associated with increased bone turnover and osteoporosis, particularly in postmenopausal women and aging populations. In addition to its impact on bone health, prolonged hypercalciuria can contribute to renal dysfunction and other metabolic imbalances, complicating patient management [3].

Diagnosing and treating IH remains a challenge for clinicians. This article will cover the pathophysiology, clinical implications, diagnostic techniques, therapeutic approaches, and current research related to IH.

## Review

Pathophysiology of stone formation

Supersaturation and Nucleation

Urinary supersaturation is the primary driving force for crystal nucleation and stone formation. In IH, elevated urinary calcium levels increase the likelihood of calcium oxalate (CaOx) and calcium phosphate (CaP) precipitation [2]. Supersaturation with respect to CaOx is influenced by urinary calcium and oxalate concentrations, whereas CaP supersaturation is determined by calcium, phosphate, and urinary pH [3]. Crystallization occurs when solute concentration exceeds its solubility threshold, leading to the formation of microscopic crystal nuclei. These nuclei can either be excreted in urine or adhere to renal surfaces, initiating stone formation [4].

Randall's Plaque and Interstitial Calcium Deposits

Randall's plaques, subepithelial deposits of interstitial calcium phosphate (apatite), serve as nucleation sites for stone formation [5]. These plaques originate in the basement membrane of the thin loops of Henle, where calcium phosphate crystallization occurs in the interstitium before breaching the urothelium [6]. Clinical evidence suggests that CaOx stones frequently form as overgrowths on Randall's plaque. Plaque formation is influenced by urinary supersaturation, reduced renal calcium reabsorption, and interstitial mineralization [7].

Tubular Plugging and Crystal Retention

In some stone formers, crystallization occurs within the renal tubules, leading to Bellini duct plugging. Tubule plugging involves intraluminal precipitation of calcium phosphate, which extends into the collecting ducts and renal papillae [8]. Patients with CaP stones exhibit extensive intratubular mineralization, often accompanied by nephron loss and cortical fibrosis [9]. When tubule plugs protrude into the urinary space, they can act as nucleation sites for stone formation. Overgrowths of CaOx or brushite (CaHPO_4_) can develop on exposed crystalline surfaces, leading to stone enlargement [10].

Role of Urinary Inhibitors and Promoters

Urinary composition plays a crucial role in modulating crystal formation. Certain molecules act as inhibitors, reducing crystal aggregation and promoting stone dissolution. Key inhibitors include citrate, which binds calcium, reducing supersaturation and inhibiting crystal nucleation. Magnesium competes with calcium for oxalate binding, decreasing CaOx precipitation and glycoproteins (e.g., osteopontin, Tamm-Horsfall protein) and preventing crystal adhesion to renal epithelium [11]. Conversely, promoters of stone formation include high urinary calcium, low citrate levels, and increased urinary oxalate. Urinary pH also plays a role, as higher pH promotes CaP stone formation, whereas acidic urine favors uric acid crystallization [12].

Crystal Aggregation and Stone Growth

Once crystals form, they can either be flushed out in urine or aggregate into larger concretions. Stone growth depends on the balance between crystal retention and dissolution. Factors influencing aggregation include urinary stasis, tubular injury, and urothelial dysfunction [13].

Pathophysiology of idiopathic hypercalciuria

IH results from a complex dysregulation of calcium homeostasis, involving interactions between renal calcium handling, intestinal absorption, and skeletal turnover. These factors contribute to excessive urinary calcium loss, increasing the likelihood of kidney stone formation and bone demineralization.

Through the interaction of the renal tubules, gastrointestinal tract, and bone mineral metabolism, the human body maintains strict regulation of calcium. The renal tubules of the kidneys reabsorb calcium after it has been filtered by the glomeruli [14]. About 99% of the filtered calcium is reabsorbed, while the remaining 1% is eliminated in the urine. Numerous factors, such as food intake, hormonal modulation, and renal function, affect calcium excretion in the urine [15]. Excessive excretion of calcium in the urine results from a disruption in calcium homeostasis, which leads to IH. This syndrome has been explained by several mechanisms, as discussed below.

Renal Handling of Calcium

The kidneys play a critical role in calcium balance, filtering and reabsorbing approximately 98-99% of the calcium that passes through the glomeruli. In IH, this finely tuned process is disrupted, leading to increased urinary calcium excretion.

Patients with hypercalciuria may experience increased renal calcium losses through two mechanisms: elevated filtering load of calcium or decreased calcium reabsorption in tubules. The pathophysiological mechanism that underlies IH is mainly related to decreased calcium reabsorption in renal tubules.

Patients with IH and those with normal urine calcium levels have serum concentrations of ultrafilterable calcium that overlap during both fed and fasting periods while following a controlled diet. In a study conducted by Worcester et al., seven control patients and 10 patients with IH consumed a regulated diet for three days following a fast. There was no difference in the filtered calcium load or ultrafilterable calcium between the two groups during fasting or post-meal status. However, urine fractional calcium reabsorption in people with IH was much lower during fasting or after meals, indicating a renal tubular defect to preserve calcium [16]. Nevertheless, when calcium was administered intravenously, other researchers were unable to demonstrate any appreciable variations in the renal tubular reabsorption of calcium between normocalciuric and hypercalciuric patients [17]. These contradictory results emphasize the possibility that individuals with IH are a diverse group with varying underlying pathophysiological mechanisms.

The root cause of "renal leak" is still unknown. The underlying defect appears to be reduced calcium reabsorption in the renal tubules, particularly in the distal nephron. Several molecular mechanisms have been implicated, including dysregulation of calcium transport proteins, such as transient receptor potential vanilloid subfamily 5 (TRPV5) and sodium-calcium exchangers [15,16]. Studies show that individuals with IH have significantly lower calcium reabsorption, even in the presence of normal serum calcium levels [17]. Recent research suggests abnormal paracellular calcium transport in the proximal tubule may also contribute to hypercalciuria. Claudin-2, a tight junction protein involved in passive calcium transport, has been found to be overexpressed in some patients with IH, potentially exacerbating urinary calcium loss. Additionally, sodium transport mechanisms, particularly the sodium-chloride cotransporter (NCC), may indirectly influence calcium handling by altering sodium gradients that impact calcium reabsorption [18,19].

Endogenous lithium clearance, a measure of proximal tubule reabsorption, can be used to quantify the amount of calcium that is delivered from the proximal tubule into more distal nephron segments. Using this method, it was found that patients with IH had higher distal calcium delivery than those with normal calcium excretion [18]. This finding indicates that the proximal nephron segments contribute to the increase in urine calcium excretion in these patients.

Intestinal Calcium Absorption

In individuals with normal urine calcium excretion, intestinal calcium absorption increases as food intake increases; on average, both men and women absorb 20% of their dietary calcium [19]. In contrast, individuals with IH absorb about 30% of their dietary calcium [20]. Increased calcium absorption in the small intestine through both a paracellular passive process and an active transcellular process in the duodenum and upper jejunum via transient receptor potential vanilloid subfamily member 6 (TRPV6) may result in increased dietary calcium absorption, thereby raising serum calcium, leading to hypercalciuria [21]. Increased calcium absorption in patients with IH may be due to elevated tissue vitamin D receptor (VDR) expression, elevated serum calcitriol levels, or a combination of these variables, as the vitamin D hormone system enhances epithelial calcium transport. Additionally, increased expression of VDR in the duodenal mucosa has been documented in IH patients, supporting the hypothesis that vitamin D signaling plays a crucial role in disease pathogenesis. People with IH typically have greater serum calcitriol levels than people with normal calcium excretion in their urine, which may indicate that IH is primarily caused by the activation of the vitamin D hormone system [22].

Bone Resorption

Another potential mechanism is increased bone resorption. In individuals with IH, increased osteoclastic activity could lead to elevated calcium levels in the circulation, which is subsequently filtered and excreted by the kidneys. Many patients with IH exhibit evidence of excessive bone resorption, which contributes to reduced bone mineral density (BMD) and increased fracture risk. Elevated levels of bone turnover markers, including N-terminal telopeptide and C-terminal telopeptide, indicate heightened osteoclastic activity in these individuals [23]. This increased bone resorption appears to be driven by an exaggerated response to parathyroid hormone (PTH) despite normal circulating PTH levels [24]. Recent findings suggest that calcium-sensing receptor (CaSR) dysfunction may also contribute to bone loss in IH. The CaSR regulates calcium homeostasis in bones, kidneys, and the parathyroid glands. Alterations in CaSR activity may disrupt the balance between osteoblastic and osteoclastic activity, leading to excessive calcium mobilization from bone stores and, consequently, hypercalciuria [25]. Most patients with IH appear to have impaired renal calcium reclamation and increased intestinal calcium absorption. However, in balance studies, urine calcium output frequently surpasses gastrointestinal calcium absorption, and people with IH may excrete more calcium than they consume when their dietary calcium intake is extremely low [23]. These findings imply that when patients are given a low-calcium diet to prevent stones, IH will result in bone damage. Numerous studies have demonstrated decreased BMD and an elevated risk of fractures among people with IH, which is consistent with this theory [24].

Genetic Factors

IH may be hereditary, with a familial clustering observed in many cases. Genetic mutations affecting calcium transporters, such as *TRPV5*, as well as CaSRs, have been linked to IH. Epigenetic factors, such as DNA methylation and histone modifications, may also influence the expression of calcium-regulating genes. Environmental factors, including diet and micronutrient exposure, can modify these epigenetic markers, providing additional insights into the complexity of IH pathogenesis. IH has long been thought to have a hereditary component. An autosomal dominant transmission was presumably the cause of 47.5% of the 40 children with IH who had at least one afflicted first-degree relative [25]. Hypercalciuria was also discovered in 43% of first-degree relatives in a study of adult patients with kidney stones and IH [26]. The increased occurrence of hypercalciuria in the second and third generations strongly suggests that IH has a genetic component. Urinary calcium excretion has been linked to loci close to genes essential for bone health and magnesium metabolism, according to genome-wide association studies (GWAS) [27]. Furthermore, hypercalciuria brought on by abnormalities in renal calcium transport has been connected to mutations in genes like claudin-2 (*CLDN2*) [28]. These genetic discoveries pave the way for focused treatment strategies, which may enable precision medicine techniques that alter particular calcium handling mechanisms. Future studies should also investigate gene-environment interactions and epigenetic changes that contribute to IH.

Clinical presentation and diagnosis

Clinical Symptoms

There are various ways that IH manifests; however, many people may have no symptoms at all. The development of kidney stones is one of the most prevalent and clinically important effects of IH [15]. Increased calcium levels in the urine have a direct impact on the development of these stones, which are usually calcium oxalate. Bone demineralization brought on by persistent hypercalciuria may raise the risk of fractures and bone discomfort [29]. Imaging tests can identify the disorder known as nephrocalcinosis, which is defined by the accumulation of calcium salts in the renal parenchyma. High calcium levels in the urine can cause recurrent urinary tract infections in certain people, which may act as a nidus for bacterial growth.

Diagnostic Approach

**Figure 1:**
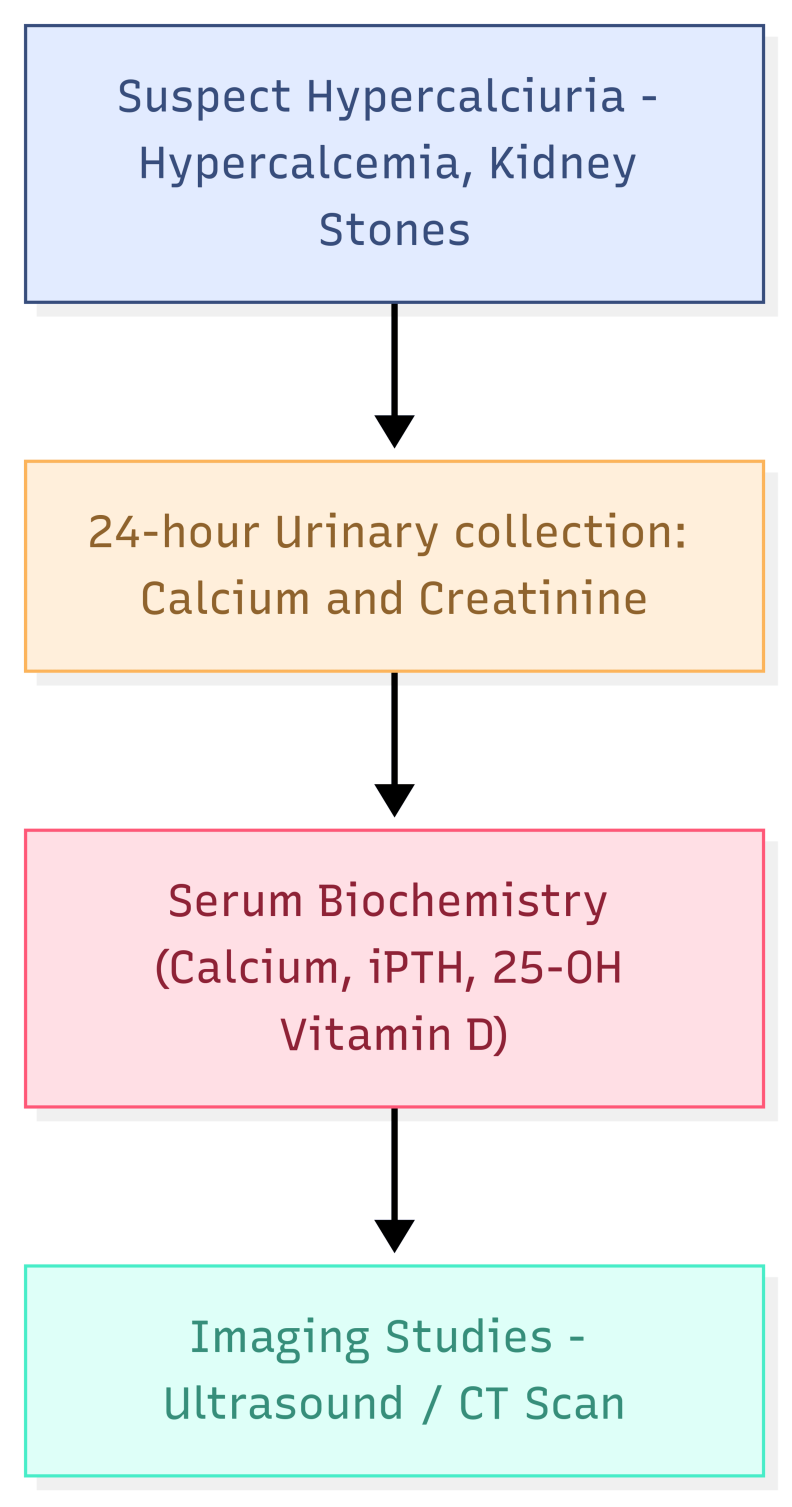
Diagnostic approach for idiopathic hypercalciuria This figure illustrates the stepwise diagnostic approach for IH, including laboratory tests and imaging. iPTH: parathyroid hormone; CT: computed tomography Image credits: Vishal Agarwal, Dayanidhi Meher

The diagnosis of IH involves ruling out secondary causes of hypercalciuria and identifying elevated calcium excretion. Figure 1 depicts the diagnostic approach for IH.

The first step in the diagnostic workup typically includes a 24-hour urine collection to measure 24-hour urinary calcium excretion. A calcium excretion of more than 4 mg/kg body weight per day in adults or more than 4.5 mg/kg in children suggests hypercalciuria [30]. The next step includes the exclusion of secondary causes of hypercalciuria, such as primary hyperparathyroidism, vitamin D excess, and malignancy must be excluded through laboratory testing (serum calcium, parathyroid hormone (PTH), and vitamin D levels) and imaging studies. In patients with a history of nephrolithiasis, imaging studies such as ultrasound, X-ray, or CT scans may be useful in assessing renal damage or nephrocalcinosis. Conditions such as hypercalcemia of malignancy, primary hyperparathyroidism, and certain medications (e.g., loop diuretics) should be considered in the differential diagnosis of IH.

Management

Conservative Management

Increasing fluid intake is one of the most important aspects of managing IH. Adequate hydration helps dilute the urine, reducing calcium supersaturation and the risk of stone formation. Urine supersaturation would be reduced by a urine flow of 100 mL/hour during fasting and nighttime and 125 mL/hour during feeding. Fluid intakes of 140 mL/hour while fasting and overnight and 165 mL/hour when fed would be predicted to attain this urine flow, given an estimate of 0.9 L/day (40 mL/hour) insensible water losses among patients who are not exposed to an extreme climate [31]. To achieve at least 2 L of urine per day, a high daily fluid intake (>3 L on average) is recommended; fluid intakes of ≥3.5 L/day are advised to reach urine volumes of ≥2.5 L/day, with adjustments for higher fluid needs based on work, climate, and lifestyle.

A balanced diet with an adequate calcium intake is essential. Despite the increased urinary calcium excretion, calcium supplementation is not typically recommended, as it may worsen hypercalciuria. A low-sodium diet is also important, as sodium increases calcium excretion in the urine. For hypercalcemic patients, dietary calcium intake should be sufficient (≥1,200 mg), and sodium intake should ideally be limited to less than 1.5 g (65 mmol) daily as advised by the Centres for Disease Control and Prevention for the adult population in the United States [32]. This low-sodium diet may lower calcium levels in the urine and preserve or improve bone mineral storage. Weight management through diet and exercise is recommended, particularly for patients who are overweight or obese. Physical activity helps maintain bone density and may reduce the risk of kidney stone formation.

Pharmacological Treatment

Thiazide diuretics are the cornerstone of treatment in IH. These medications reduce urinary calcium excretion by increasing calcium reabsorption in the distal convoluted tubule of the kidney [33]. Thiazides are effective in preventing kidney stones and reducing urinary calcium levels. They are typically prescribed at low doses and up-titrated gradually to minimize side effects such as hypokalemia and hyperglycemia [34]. Potassium or sodium citrate can help increase urinary citrate levels. Citrate binds to calcium in the urine, reducing the formation of calcium-based stones. Citrate supplementation may be particularly beneficial in patients with a history of nephrolithiasis. When citrate excretion is greater than 400 mg/day, the upper 95% CI for the relative risk ratio of developing a stone is less than 1 for both sexes, and a gradually higher risk of stone formation is associated with reduced levels of citrate excretion [35]. Therefore, in patients with idiopathic calcium stones, treatment with potassium citrate or another oral potassium-based alkali should be utilized to raise urine citrate levels to >400 mg daily and lower the risk of stone formation.

Surgical or Interventional Treatment

In patients with symptomatic kidney stones or nephrocalcinosis, surgical intervention may be required. Procedures such as lithotripsy, ureteroscopy, or percutaneous nephrolithotomy can be used to remove large stones. Nephrectomy may be indicated in severe cases with significant renal damage.

Complications and long-term outlook

The most common long-term complication of IH is the development of kidney stones. Recurrent stone formation is a significant concern, leading to chronic kidney damage, renal function decline, and nephrocalcinosis. Osteoporosis and osteopenia are potential complications due to chronic calcium loss. Decreased BMD is common in individuals with untreated IH, and these patients are at increased risk for fractures. The recurring nature of kidney stones can lead to a decreased quality of life and significant psychological distress. Pain, anxiety about stone recurrence, and frequent hospital visits may adversely affect mental well-being [36].

Prognosis

The prognosis for people with IH is generally good, but in order to avoid consequences such as nephrolithiasis, osteoporosis, and nephrocalcinosis, careful long-term surveillance is necessary. Because IH has been linked to bone demineralization, it is advised to periodically check BMD in addition to preserving renal function, particularly in patients with a history of fractures or persistent hypercalciuria [37]. Frequent 24-hour urine calcium tests are essential for assessing the effectiveness of treatment and compliance with dietary changes. Comprehensive metabolic evaluations, which include measurements of urine citrate and oxalate levels, may help patients with recurring kidney stones customize preventive measures.

With appropriate treatment, patients with IH can maintain normal renal function and quality of life. Early detection and treatment are essential to prevent stone formation and preserve kidney function.

Limitations

IH presents several challenges in both clinical practice and research. One of the main difficulties is the lack of a universally accepted definition, as different studies use varying criteria to diagnose the condition. This inconsistency makes it hard to compare research findings and establish clear guidelines for clinicians. Additionally, while genetics and environmental factors contribute to hypercalciuria, the precise interaction between these influences remains unclear. The role of diet, gut microbiota, and other metabolic factors is still being explored, and current understanding is incomplete.

Another key limitation is the shortage of long-term studies assessing how IH progresses over time. While we recognize its association with kidney stones and bone loss, there is limited data on the broader health implications, including its potential impact on cardiovascular health. Treatment options, such as thiazide diuretics and citrate therapy, have shown promise, but most studies on these interventions are small-scale and short-term, leaving questions about their long-term safety and effectiveness. Additionally, much of the existing research focuses on middle-aged adults, while children and older adults remain underrepresented, making it difficult to tailor treatment strategies for these populations.

Recommendations

Several steps should be taken to improve both research and patient care. First, we need a standardized approach to diagnosing IH, including consistent urine calcium measurement protocols and clearer criteria for defining abnormal calcium excretion. This would allow for better comparison of studies and more reliable treatment recommendations. Additionally, a more personalized approach to risk assessment, taking into account genetic factors, dietary habits, and other metabolic markers, could help identify patients at higher risk for complications and guide early intervention.

Since hypercalciuria affects multiple systems in the body, a collaborative approach to care is essential. Endocrinologists, nephrologists, urologists, and dietitians should work together to create individualized management plans that go beyond calcium restriction alone. While reducing dietary calcium intake was once considered a standard recommendation, we now understand that other dietary factors, such as sodium, protein, and vitamin D, play significant roles in calcium metabolism. More research is needed to refine dietary guidelines and explore non-pharmacological approaches, such as gut microbiome modulation, which may offer new ways to manage calcium balance.

Future directions

Looking ahead, there is a great need for research that delves deeper into the genetic and molecular mechanisms driving IH. Advances in genomics and metabolomics could help identify individuals at higher risk and lead to the development of targeted therapies that go beyond current treatment options. Additionally, the gut-kidney axis is an exciting area of exploration, as changes in intestinal calcium absorption and gut microbiota composition may play a bigger role than previously thought. Understanding these interactions could pave the way for novel treatment approaches, such as probiotics or dietary modifications that influence calcium handling in the body.

Newer pharmacological treatments that specifically target renal calcium transporters and bone metabolism are also worth exploring. While thiazides have been the cornerstone of pharmacological management, they are not without side effects, and alternative therapies may provide safer and more effective long-term solutions. In addition, more research is needed to understand how IH affects children and older adults. Studying how the condition progresses in these populations could help identify the best strategies for preventing complications like kidney stones and bone loss from an early stage.

Finally, long-term studies that track patients over several decades would be invaluable. By following individuals with IH over time, we could gain a clearer understanding of how different treatment approaches impact kidney stone formation, fracture risk, and overall metabolic health. With this knowledge, we can refine our clinical guidelines and ensure that patients receive the most effective, evidence-based care possible.

## Conclusions

IH is a complex condition with important clinical implications, particularly in the formation of kidney stones and its impact on bone health. Managing this condition effectively requires a well-rounded approach that includes dietary adjustments, medication when necessary, and regular monitoring to reduce complications. While current treatment strategies aim to control calcium balance and prevent kidney stones, significant knowledge gaps remain about the underlying mechanisms driving this condition. Research is continually evolving, shedding light on genetic factors, metabolic pathways, and even the role of gut microbiota in calcium regulation.

Despite these advancements, challenges remain, particularly in standardizing diagnosis and determining the best long-term management strategies for different patient groups, including children and older adults. There is a growing need for more research into how lifestyle factors, genetics, and emerging therapies, such as microbiome-based interventions, can be used to create more personalized treatment approaches. Looking ahead, continued progress in precision medicine may allow for better risk assessment and more targeted therapies, ultimately improving patient outcomes. Long-term studies will be key to refining current guidelines and ensuring that treatment is both effective and sustainable. With ongoing research and a multidisciplinary approach, we can continue to improve the management of IH, helping to reduce the burden of kidney stones, preserve bone health, and enhance the overall well-being of affected individuals.
